# Dimethyl celecoxib sensitizes gastric cancer cells to ABT‐737 *via* AIF nuclear translocation

**DOI:** 10.1111/jcmm.12913

**Published:** 2016-07-04

**Authors:** Bo Zhang, Youyou Yan, Yangling Li, Dan Zhang, Jianmei Zeng, Linling Wang, Mimi Wang, Nengming Lin

**Affiliations:** ^1^ Laboratory of Clinical PharmacologyHangzhou First People's HospitalNanjing Medical UniversityHangzhouZhejiangChina; ^2^ Laboratory of Clinical PharmacologyHangzhou Translational Medicine Research CenterHangzhou First People's HospitalHangzhouZhejiangChina; ^3^ Department of Clinical PharmacyHangzhou First People's HospitalHangzhouZhejiangChina; ^4^ Institute of PharmacologyCollege of Pharmaceutical SciencesZhejiang Chinese Medical UniversityHangzhouZhejiangChina

**Keywords:** ABT‐737, dimethyl celecoxib, gastric cancer, AIF

## Abstract

Gastric cancer is the fourth most common cancer in the world. The clinical applications of both chemotherapy and targeted drugs are limited because of the complexity of gastric cancer. In this study, sulforhodamine B, colony formation assay, 4',6‐diamidino‐2‐phenylindole (DAPI) stain, flow cytometry were used to determine the *in vitro* cytotoxicity, apoptosis and mitochondrial membrane potential of gastric cancer AGS and HGC‐27 cells before and after treatment. Real‐time PCR and Western blot were used to analyse the mRNA transcription and protein expression respectively. Confocal microscopy was used to determine the localization of target protein within the cells. Treatment with the combination of ABT‐737 and 2,5‐dimethyl‐celecoxib (DMC) showed strong synergistic effect in both AGS and HGC‐27 cells. Moreover, DMC would not influence the intracellular prostaglandin E2 (PGE2) level, thus lacking the toxicity profile of celecoxib. Interestingly, given the significant synergistic effect, combination treatment did not affect the protein expression of BH‐3 proteins including Puma, Noxa and Bim. In combination treatment, cell apoptosis was found independent of caspase‐3 activation. The translocation of apoptosis‐inducing factor (AIF) from mitochondrion to nuclear was responsible for the induced apoptosis in the combination treatment. Taken together, this study provided a novel combination treatment regimen for gastric cancer. Furthermore, the existence of caspase‐independent apoptotic pathway induced by treatment of ABT‐737 was not yet seen until combined with DMC, which shed light on an alternative mechanism involved in Bcl‐2 inhibitor‐induced apoptosis.

## Introduction

Gastric cancer (GC) is the fourth most common cancer and the second leading cause of cancer‐related death worldwide 1. Gastric cancer is often diagnosed at advanced stage in most patients and the prognosis is yet very poor, with an average of 20% 5‐year overall survival 2, 3. Despite tremendous efforts in the worldwide clinical trials of adjuvant chemotherapy treating GC, it is still controversial in GC standard chemotherapy regimen because of the complexity of GC stage 4. Therefore, exploring efficacious compounds and chemotherapy regimens for GC are predominant problems which scientists will urgently set out to solve in the near future.

Overexpression of anti‐apoptotic Bcl‐2 family proteins such as Bcl‐2, Bcl‐xL and Mcl‐1 is closely associated with tumor initiation, progression and chemoresistance 5, 6. To abrogate these pro‐survival proteins, Bcl‐2/Bcl‐xL inhibitor ABT‐737 and its orally available compound ABT‐263 are developed, and has achieved satisfied results in both preclinical experiments and clinical trials 7. However, either ABT‐737 or ABT‐263 lacks the binding affinity to Mcl‐1, which makes the Mcl‐1 up‐regulated cancer cells resistant to ABT‐737 and ABT‐263, thus limiting their clinical applications as a single agent 8, 9. Therefore, diminishing the expression of Mcl‐1 has become an effective strategy to re‐sensitize resistant cancer cells to ABTs, and therefore can potentially broaden the clinical application of ABTs 10, 11.

Cyclooxygenase‐2 (COX‐2) is an essential factor in cancer promotion and progression. Major efforts have been devoted to target COX‐2 to reduce tumor growth, invasion and metastasis 12, 13. However, the clinical application of COX‐2 inhibitors, such as celecoxib, was substantially limited because of their detrimental cardiovascular side effects at pharmaceutical doses 14.Recently, it is found that celecoxib can exert its anticancer effects through COX‐2‐independent pathways, implying a promising alternative strategy using celecoxib analogues 15. 2,5‐dimethyl‐celecoxib (DMC) is an analogue of celecoxib, but lacks COX‐2‐inhibitory activity^16^. Studies have demonstrated that aggravated endoplasmic reticulum stress (ER stress), anti‐angiogenisis, induction of apoptosis and inhibition of cell cycle progression are independently involved in the antitumor activity of DMC 17, 18, 19, 20. Notably, DMC caused cell death is found to be associated with down‐regulation of Mcl‐1 protein expression in leukaemic cells 17, which inspires us that DMC can potentially synergize with ABT‐737 through the regulation of Mcl‐1 level.

The aim of this study is to investigate whether DMC would sensitize GC cells to ABT‐737 by regulating the Mcl‐1 level. We here showed that DMC exhibited stronger synergistic effect than celecoxib in combination with ABT‐737 in GC cells. The apoptosis induced by combination treatment of DMC and ABT‐737 was independent of either ER stress or BH3‐only proteins, and was closely correlated with the Mcl‐1 protein level. Notably, caspase pathway activation was not a predominant event in the combination treatment‐induced apoptosis. Instead, the translocation of apoptosis‐inducing factor (AIF) from cytosol to nucleus determined cell fate in such apoptosis. In conclusion, our data not only suggested a potentially effective chemotherapy regimen for GC, but also elucidated a caspase‐independent apoptosis pathway that involved in DMC and ABT‐737 combination treatment.

## Materials and methods

### Materials and cell culture

F12, RPMI‐1640 medium and foetal bovine serum (FBS) were purchased from Gibco, BRL (Grand Island, NY, USA). PGE2 ELISA kit ADI‐900‐001 was purchased from Enzolifesciences (Farmingdale, NY, USA). Hoechst 33258 was purchased from Sigma‐Aldrich (St Louis, MO, USA). The Annexin V‐FITC Apoptosis Kit was purchased from Becton Dickinson (Franklin Lakes, NJ, USA). Mitochondrial Membrane Potential Assay Kit was purchased from Beyotime Biotechnology (Jiangsu, China). The primary antibodies against ATF‐4, Chop, Bax, Bcl‐2, Mcl‐1, Puma, Noxa, Bim, Bak, procaspase‐3, active caspase‐3, procaspase‐9, poly ADP‐ribose polymerase (PARP) and β‐actin were purchased from Abcam Inc. (Cambridge, MA, USA). Human gastric carcinoma cell line AGS and HGC‐27 cells were purchased from Shanghai Institutes for Biological Sciences, CAS (Shanghai, China). Cells were cultured with F12 or RPMI 1640 medium containing 10% FBS and 1% penicillin/streptomycin at 37°C, 5% CO_2_ humidified atmosphere. Both ABT‐737 and DMC were dissolved in Dimethyl sulfoxide (DMSO) at the concentration of 100 mM.

### Cell viability assay

Cell viability was determined by sulforhodamine B (SRB) Assay. After the incubation period, cell monolayers were fixed with 10% (wt/vol) trichloroacetic acid and stained for 1 hr at 4°C. The plates were washed five times with deionized water, and then dried in an oven at 60°C. When completely dry, 50 μl of SRB was added to each well for 20 min., and then the excess dye was removed by washing five times with 1% (vol/vol) acetic acid, followed by further drying in an oven at 60°C. The protein‐bound dye was dissolved in 10 mM Tris‐base solution for OD determination at 510 nm by a Multiskan Spectrum (Thermo Electron Corporation, Marietta, OH, USA). Cell viability was calculated for each well as [OD510 treated cells/OD510 control cells] × 100%. Assays were performed on three independent experiments.

### Calculation of combination Index

The synergistic effects of ABT‐737 and DMC/Celecoxib were quantitatively assessed by calculation of combination index (CI) using Calcusyn^®^ (Great Shelford, Cambridge, UK). CI < 1, CI = 1 and CI > 1 indicate synergism, additive effect and antagonism respectively.

### Colony formation assay

Both AGS and HGC‐27 cells were seeded into 6‐well plates at the density of 1000/well and incubated for 48 hrs. Cells were then treated with indicated concentrations of compounds. After a 10‐day culture period, cells were fixed with 4% paraformaldehyde for 15 min. and stained with Giemsa solution for another 15 min. The visible colonies were photographed by ChemiDoc XPS system (Bio‐Rad, Hercules, CA, USA).

### *In vitro* PGE2 assay

*In vitro* PGE2 assay was performed as described by the manufacturer's instruction. Briefly, AGS and HGC‐27 cells were seeded in 10‐cm dish at the density of 1 × 10^6^ cells/dish and cultured for 24 hrs. Cells were then treated with celecoxib or DMC at the concentration of 5 μM for another 24 hrs. Cells were collected and washed with PBS twice before undergoing frozen‐thawed cycles for six times between liquid nitrogen and 37°C water bath. The resultant was centrifuged at 14,500 × g. for 30 min. Portion of the collected supernatant was used to measure the protein concentration by bicinchoninic acid (BCA) assay. The remaining supernatant underwent PGE_2_ determination. PGE_2_ concentration was normalized by protein concentration.

### Detection of mitochondrial membrane potential

Mitochondrial membrane potential was visualized by 5,5′,6,6′‐tetrachloro‐1,1′,3,3′ tetraethyl‐imidacarbocyanine iodide (JC‐1) stain. Cells were seeded into 6‐well plates at the density of 3 × 10^3^/well, and cultured for 24 hrs. After treatment, cells were collected, washed with PBS and incubated with JC‐1 for 15 min. at 37°C. After washing off the dye, cells were immediately analysed using a flow cytometry (Becton Dickinson). Assays were performed on three independent experiments.

### Hoechst 33342 stain

Both AGS and HGC‐27 cells (2 × 10^4^ cells/well) were cultured in 24‐well plates. After treatment, cells were fixed with 4% paraformaldehyde for 20 min., and stained with Hoechst 33342 for 20 min. at 37°C. After wash with PBS, cells were observed under a fluorescence microscope (Nikon, Ti‐E, Japan).

### Apoptosis assay

Exponentially growing cells were seeded in 6‐well plates (4 × 10^4^/well) and cultured overnight in a 5% CO_2_ atmosphere at 37°C. After treatment with indicated compounds for 24 hrs, cells were harvested and washed with PBS. Cells were then stained with Annexin V‐FITC Apoptosis Kit according to the manufacturer's instructions and analysed by flow cytometry (Becton Dickinson). Assays were performed on three independent experiments.

### Cytosolic calcium concentration determination

Exponentially growing cells were seeded in 6‐well plates (4 × 10^4^/well) and cultured overnight in a 5% CO_2_ atmosphere at 37°C. After treatment with indicated compounds for 6 hrs, cells were harvested and co‐cultured with Fluo‐3 AM at 37°C for 45 min. Cells were then collected and suspended in PBS, followed by flow cytometric analysis at an excitation wavelength of 488 nm.

### Silencing of gene expression with small interfering RNA

Exponentially growing cells were seeded in 6‐well plates (4 × 10^4^/well) and cultured overnight in a 5% CO_2_ atmosphere at 37°C. The medium was then replaced with Opti‐MEM I Reduced Serum Media (Gibco) containing 20.0 nM Mcl‐1 small interfering RNA (siRNA; GenePharma, Shanghai, China) and oligofectamine reagent (Invitrogen^™^; Thermofisher Scientific, Waltham, MA, USA) according to manufacturer's recommendations. Forty‐eight hours after transfection, cells were harvested or treated with DMSO vehicle, serial concentrations of ABT‐737, DMC or the combination.

### Real‐time reverse transcriptase PCR

Total RNA was extracted from sample cells with TRIzol, precipitated with isopropyl alcohol and rinsed with 70% ethanol. Single‐strand cDNA was prepared from the purified RNA using oligo (dT) priming (Invitrogen^™^; Thermofisher Scientific), followed by SYBR‐Green real‐time PCR (Qiagen, Hilden, Germany). The primers used are as follows: Mcl‐1, 5′‐GGGCAGGATTGTGACTCTCATT‐3′, 5′‐GATGCAGCTTTCTTGGTTTATGG‐3′; GAPDH, 5′‐GAGTCAACGGATTTGGTCGT‐3′, 5′‐TTGATTTTGGAGGGATCTCG‐3′.

### Western blot analysis

After treated with ABT‐737, DMC or the combination, total proteins were extracted using RIPA Lysing Buffer. An amount of 40 μg proteins were subjected to 12% SDS‐PAGE and transferred to PVDF Membrane (Bio‐Rad). The membranes were blocked with 5% non‐fat milk at room temperature for 1 hr, and then incubated with specific primary antibodies overnight at 4°C. After washing with TBST, the membranes were incubated with secondary antibodies at room temperature for another 1 hr. The protein bands were visualized by adding ECL system WBKLS0050 (EMD Millipore, Billerica, MA, USA) and analysed using Bio‐Rad Laboratories Quantity One software (Bio‐Rad). Proteins from cytosolic and nuclear extracts were separated by using Nuclear/Cytosol Fractionation Kit (Biovision, Milpitas, CA, USA).

### Immunofluorescence

AGS and HGC‐27 cells were plated into Sigma Nunc^®^ Lab‐Tek^®^ II chambered coverglass 8 wells Sigma‐Aldrich at 10,000 cells per chamber in complete medium and incubated for 24 hrs before use. The medium was replaced with ABT‐737, DMC or the combination and then cultured for 4 hrs. Cells were then rinsed with PBS twice before fixation by 4% formaldehyde for 20 min. at room temperature. Cells were then rinsed with PBS for three times and permeabilized by 0.2% Triton X‐100 for 10 min. After washing with PBS, cells were blocked by 5% BSA for 30 min. and incubated with primary antibody overnight at 4°C. Cells were washed by PBS and incubated with secondary antibody (Goat Anti‐Rabbit IgG H&L, Alexa Fluor^®^ 594; Abcam Inc.) for 30 min. The slides were sealed with coverglasses using ProLong^®^ Gold antifade reagent with DAPI (Invitrogen^™^; Thermofisher Scientific), and immediately observed by confocal microscope (Leica SP8; Leica, Wetzlar, Germany). Apoptosis‐inducing factor was visualized by excitation at 552 nm and its fluorescence emission was observed using a 570–710 nm band‐pass filter. DAPI was excited at 405 nm and the emission was detected ranging from 420 to 540 nm.

### Statistical analysis

The results are expressed as the mean ± S.D. of at least three independent experiments. Differences between means were analysed using Student's *t*‐test and were considered statistically significant when *P* < 0.05. Graphs were prepared using SigmaPlot 12.0 software (Systat Software, Inc. San Jose, CA, USA).

## Results

### Cytotoxicity of ABT‐737 and DMC/Celecoxib in human GC cells

**Figure 1:**
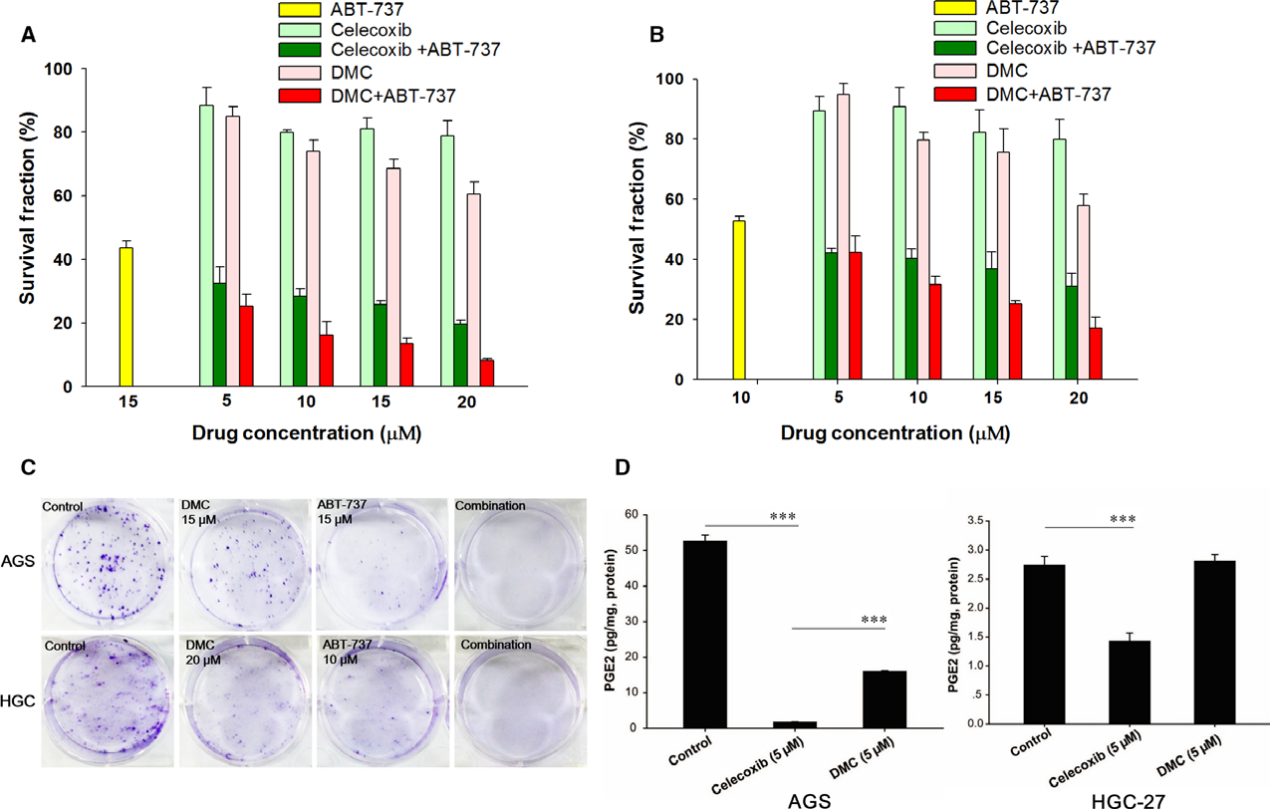
The inhibitory effect of celecoxib, DMC, ABT‐737 or the combination on two human gastric cancer cell lines. AGS (**A**) and HGC‐27 (**B**) cells were seeded in 96‐well plates and then treated with celecoxib, DMC, ABT‐737 or the combination as indicated. Treatment was washed off after 24 hrs and cell viability was calculated using SRB assay. (**C**) AGS and HGC‐27 cells were seeded in 6‐well plates and cultured for 48 hrs and co‐cultured with DMC, ABT‐737 or the combination for another 10 days. Cell colonies were fixed by formalin, and visualized by Giemsa stain. (**D**) The cytosolic PGE2 concentration (normalized by protein concentration) in the presence of celecoxib (5 μM) or DMC (5 μM) was determined using Elisa assay according to the manufacture's instruction. Each bar represented the mean ± S.D., ***P < 0.001.

The cytotoxicity of ABT‐737, DMC/Celecoxib or the combination was determined by SRB assay in two human GC cell lines AGS (Fig. 1A) and HGC‐27 (Fig. 1B) cells. In comparison with ABT‐737 or DMC/Celecoxib mono‐treatment, the combination of ABT‐737 and DMC/Celecoxib exhibited much stronger anti‐proliferation effects in both AGS and HGC‐27 cells. At the same concentrations, the combination of ABT‐737 and DMC showed higher cytotoxicity than the combination of ABT‐737 and Celecoxib in both cell lines. Combination index values were calculated by Calcusyn Software (Tables 1 and 2). The CI values from ABT‐737 and DMC were literally lower than that of ABT‐737 and Celecoxib. Synergy (CI <0.70) or strong synergy (CI <0.30) was observed in both cell lines. In addition, the combination of DMC and ABT‐737 obviously inhibited the formation of cell colony (Fig. 1C). Interestingly, as a typical COX‐2 inhibitor, Celecoxib significantly diminished the intracellular PGE2 level. In contrast, DMC scarcely affected PGE2 level under the same treatment condition (Fig. 1D).

**Table 1:** The combination index (CI) of DMC/Celecoxib and ABT‐737 in AGS cells

DMC (μM)	ABT‐737 (μM)	CI
5	15	0.283
10	15	0.157
15	15	0.095
20	15	0.013

Celecoxib (μM)	ABT‐737 (μM)	CI
5	15	0.301
10	15	0.152
15	15	0.105
20	15	0.037

**Table 2:** The combination index (CI) of DMC/Celecoxib and ABT‐737 in HGC‐27 cells

Celecoxib (μM)	ABT‐737 (μM)	CI
5	10	0.551
10	10	0.528
15	10	0.406
20	10	0.286

DMC (μM)	ABT‐737 (μM)	CI
5	10	0.488
10	10	0.432
15	10	0.343
20	10	0.245

### ABT‐737 synergized with DMC to trigger apoptosis

**Figure 2:**
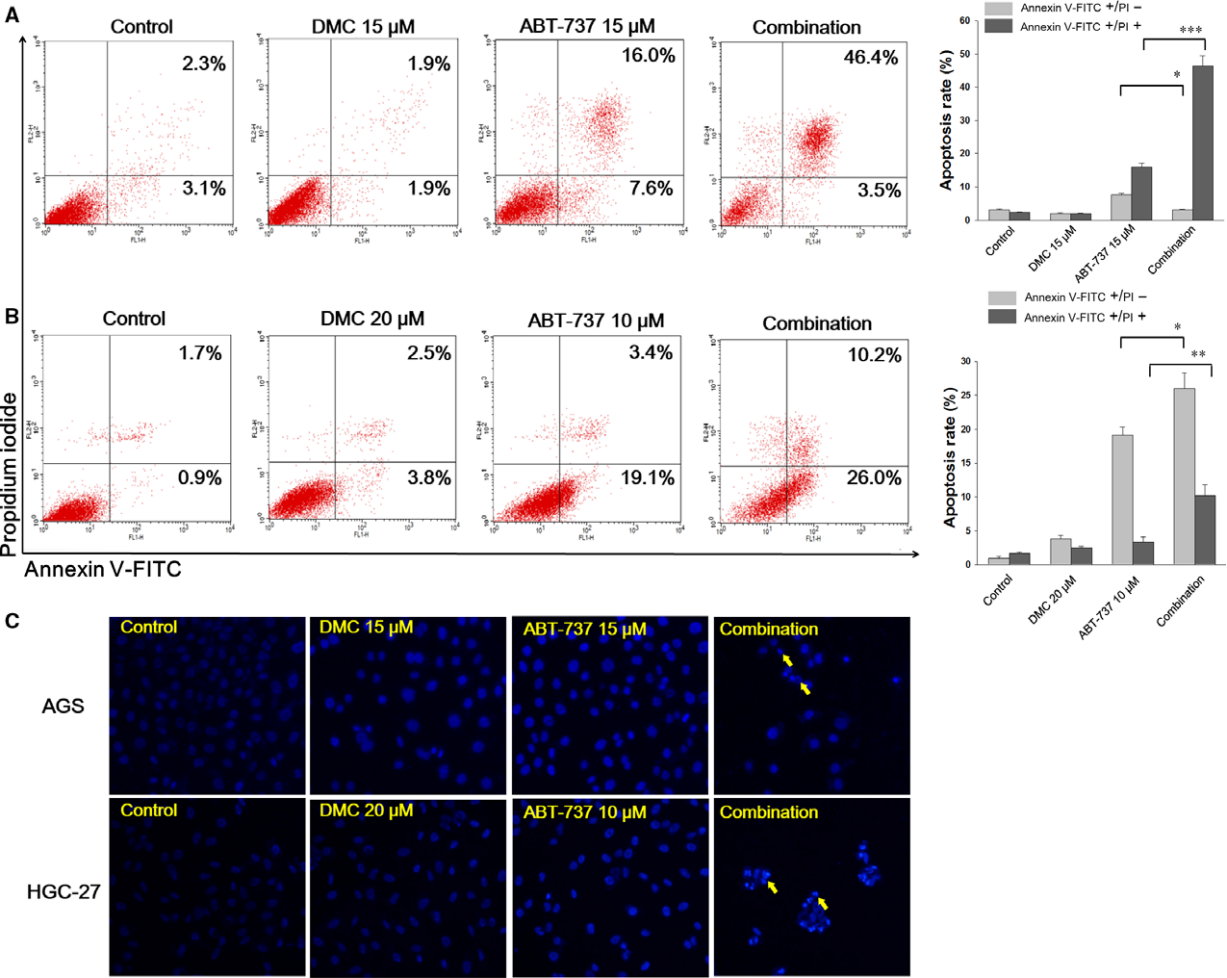
DMC, ABT‐737 and combination induced apoptosis in AGS and HGC‐27 cells. AGS (**A**) and HGC‐27 (**B**) cells were seeded in 6‐well plate and cultured for 24 hrs. Cells were treated with DMC, ABT‐737 and combination for 24 hrs before staining and followed by flow cytometric analysis. Three independent experiments were quantitatively analysed. (**C**) AGS and HGC‐27 cells were seeded in 6‐well plate and cultured for 24 hrs. Cells were treated with DMC, ABT‐737 and combination for 24 hrs before DAPI stain and observed under fluorescence microscopy. Each bar represented the mean ± S.D. *P < 0.05, **P < 0.01, ***P < 0.001.

As DMC were reported to induce apoptosis in cancer cells, the synergistic effects of ABT‐737 and DMC on apoptosis were determined in both AGS and HGC‐27 cells. Annexin V/PI staining was used to characterize the early and late apoptotic cells after cells were treated with indicated concentrations of ABT‐737, DMC or the combination for 24 hrs. In AGS cells, the mono‐treatment of ABT‐737 or DMC resulted in an overall apoptosis rate of 23% and 4% respectively (Fig. 2A). While cells were treated with the combination of ABT‐737 and DMC, the proportion of apoptotic cells increased to 50%. In HGC‐27 cells, the combination of ABT‐737 and DMC also achieved higher apoptosis rate than mono‐treatment. The treatment of ABT‐737 or DMC resulted in an overall apoptosis rate of 22% and 6% respectively (Fig. 2B), while the combination treatment induced a 36% apoptosis rate.

The morphology change in cell nucleus was observed by DAPI staining after treatment with ABT‐737, DMC or the combination for 24 hrs (Fig. 2C). Compared to untreated cells, the fluorescence intensity slightly elevated in the mono‐treated cells, indicating the condensed nucleus after mono‐treatment. In the combination treatment group, not only the cell number greatly diminished, apoptotic bodies were prevalent in the observation field (Yellow arrows in Fig. 2C).

### Mitochondrion plays a pivotal role in the apoptosis induced by the combination of ABT‐737 and DMC

**Figure 3:**
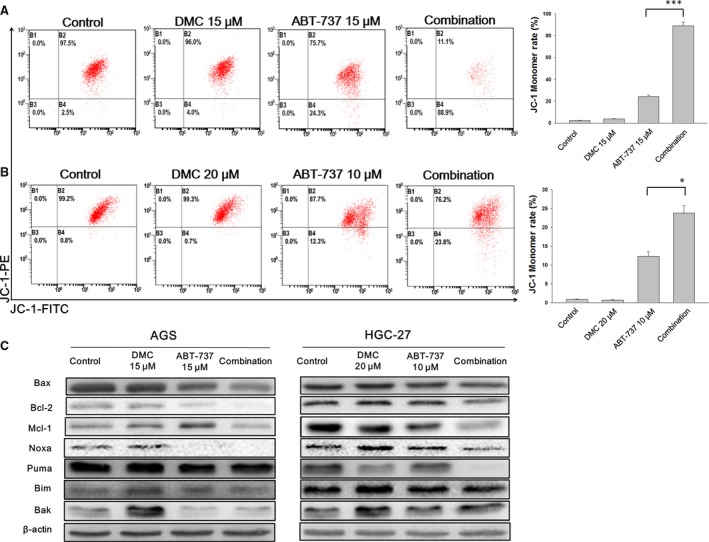
DMC, ABT‐737 and combination disrupted mitochondrial membrane potential and its regulatory proteins. JC‐1 stain was used to detect the mitochondrial membrane potential after 4 hrs treatment with indicated compounds in AGS cells (**A**) and HGC‐27 cells (**B**), followed by flow cytometric analysis. After treatment with DMC, ABT‐737 or the combination for 12 hrs, cells were lysed and total protein was collected for Western blot assay (**C**). Each bar represented the mean ± S.D. *P < 0.05, ***P < 0.001.

The mitochondrial membrane potential in the cells exposed to ABT‐737, DMC or the combination for 12 hrs was examined by JC‐1 staining. The proportion of cells shifting from red to green fluorescence suggested the cells undergoing mitochondrial depolarization. In 12 hrs‐treated AGS and HGC‐27 cells, moderate proportions of cells changed to green fluorescence in mono‐treatment groups. After combination treatment with ABT‐737 and DMC, over 80% AGS cells exhibited depolarized mitochondrial membrane potential (Fig. 3A), and more than 20% HGC‐27 cells showed significantly decreased potential (Fig. 3B).

**Figure 4:**
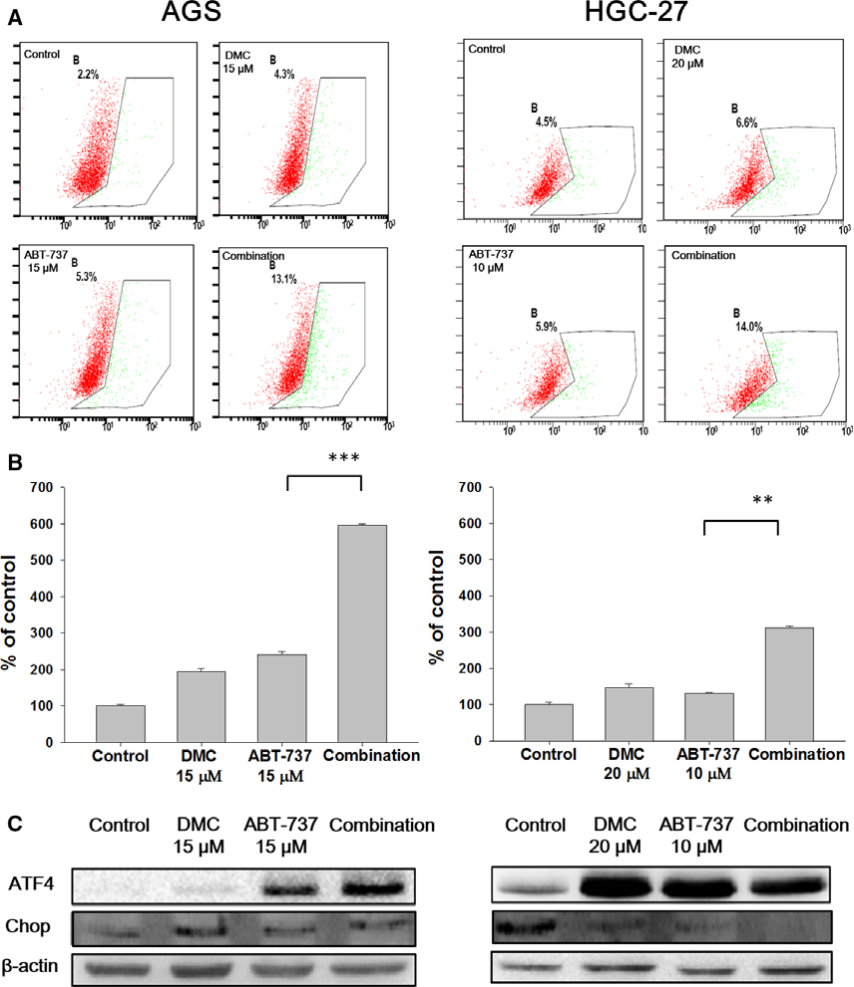
The combination treatment of DMC and ABT‐737 induced ER stress, but not the related apoptosis. Both AGS and HGC‐27 cells were treated with DMC, ABT‐737 or the combination for 4 hrs. The cytosolic calcium concentration was stained by Fluo‐3 AM, followed by flow cytometry determination (**A**) and the quantitated analysis (**B**). After treatment with DMC, ABT‐737 or the combination for 4 hrs, cells were lysed and total protein was collected for Western blot assay (**C**). Each bar represented the mean ± S.D. **P < 0.01, ***P < 0.001.

Furthermore, fundamental proteins that dictated the fate of mitochondrion including Bax, Bcl‐2, Mcl‐1, Bim, Bak, Noxa and Puma were detected (Fig. 3C), and the mean intensity of each band was quantitatively analysed (Figs S2 and S3). The Bax/Bcl‐2 expression ratio which was quantitatively analysed (Fig. S1) only showed moderate increase after combination treatment with ABT‐737 and DMC. Surprisingly, the up‐regulation of BH‐3 proteins including Puma, Noxa and Bim, was not seen after either mono‐ or combination treatment (Figs S2 and S3). In addition, another apoptosis effector Bak was found unchanged by comparing the mono‐treatment and the combination treatment. As DMC was reported to trigger ER stress‐mediated apoptosis, we then detected the cytosolic calcium concentrations and symbolic proteins indicating ER stress 21. Not surprisingly, the combination treatment of DMC and ABT‐737 triggered enhanced cytosolic calcium concentration in both AGS and HGC‐27 cells (marked in green, Fig. 4A). The combination treatment resulted in very significant increase in cytosolic calcium concentration by comparing with ABT‐737‐treated cells (Fig. 4B). Meanwhile, ATF‐4 was dramatically up‐regulated in both mono‐treated and the combination‐treated cells, suggesting the occurrence of ER stress (Fig. 4C, Fig. S3). However, the protein expression of CHOP remained unchanged during the treatment, suggesting that ER stress might not be involved in ABT‐737 and DMC‐induced apoptosis (Fig. 4C, Fig. S4).

### Mcl‐1 expression level dictates the cell fate under the combination of ABT‐737 and DMC

**Figure 5:**
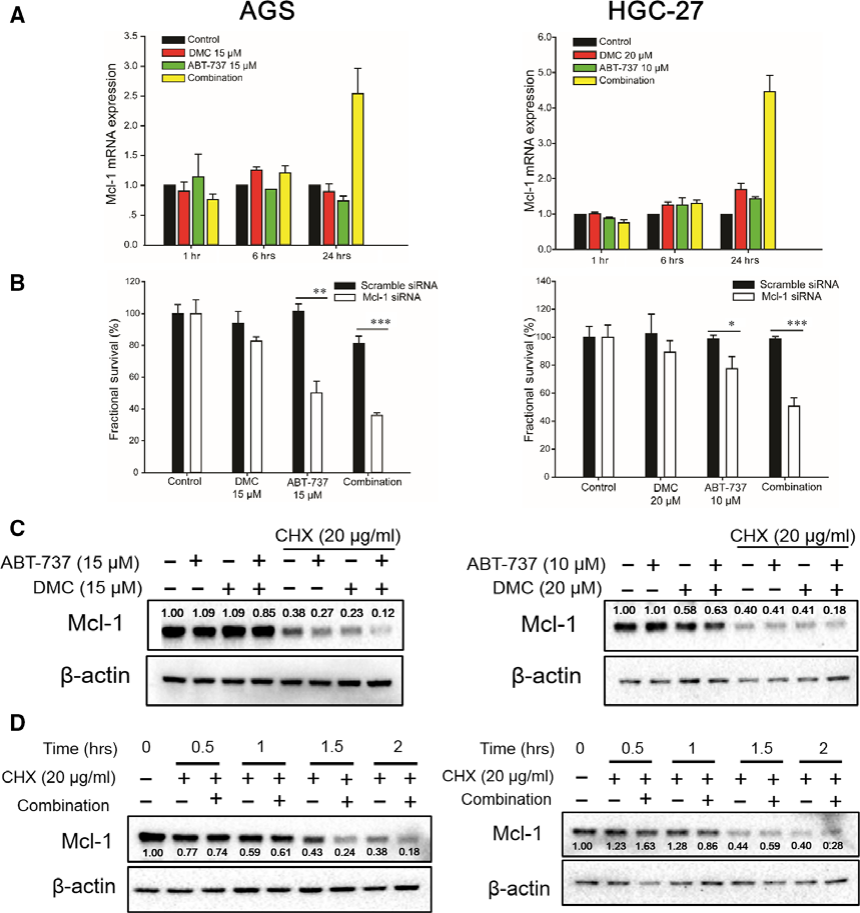
Mcl‐1 protein level determined the cytotoxicity of the combination of DMC and ABT‐737. (**A**) Cell were treated with DMC, ABT‐737 and the combination treatment for 6 or 24 hrs, then cells were lysed and total mRNA were analysed by real‐time qPCR. (**B**) Both AGS and HGC‐27 cells were transfected with Mcl‐1 siRNA and scramble siRNA and cultured for 24 hrs. Cells were then treated with indicated compounds for 24 hrs and viable cells were stained and quantitated by SRB assay. Each bar represents the mean ± S.D. (**C**) Cells were co‐treated with CHX, DMC, ABT‐737or the combination for 2 hrs before Western blot assay. (**D**) Cells were co‐treated with CHX and the combination of DMC and ABT‐737 for indicated times. Mcl‐1 protein level was determined by Western blot assay.

Constant Bcl‐2 inhibition would eventually end up with overexpression of Mcl‐1, and then acquire resistance to ABT‐737. Therefore, the elimination of Mcl‐1 could not only sensitize cells to ABT‐737, but also potentially reverse ABT‐737 resistance. Treatment with the combination of ABT‐737 and DMC for 6 hrs did not affect the Mcl‐1 mRNA level. Interestingly, Mcl‐1 mRNA level even increased after 24 hrs treatment of the combination, which probably because of the intracellular Mcl‐1 positive feedback loop (Fig. 5A). As expected, knockdown of Mcl‐1 by siRNA could sensitize both AGS and HGC‐27 cells to ABT‐737 mono‐treatment, and the combination of ABT‐737 and DMC, meanwhile having no influence on the cytotoxicity of DMC (Fig. 5B).

To understand the post‐translational process of Mcl‐1 under the treatment of ABT‐737 and DMC, protein synthesis inhibitor cycloheximide (CHX) was utilized and Mcl‐1 protein was examined. When cells were pre‐treated with 20 μg/ml of CHX for 2 hrs and co‐cultured with ABT‐737 and DMC, the combination of ABT‐737 and DMC significantly diminished the protein expression level of Mcl‐1 (Fig. 5C). Within 2 hrs duration treatment of the combination, an expedited Mcl‐1 degradation was seen at 1.5 and 2 hrs respectively in AGS and HGC‐27 cells (Fig. 5D).

### AIF translocation is responsible for the synergistic cytotoxicity

**Figure 6:**
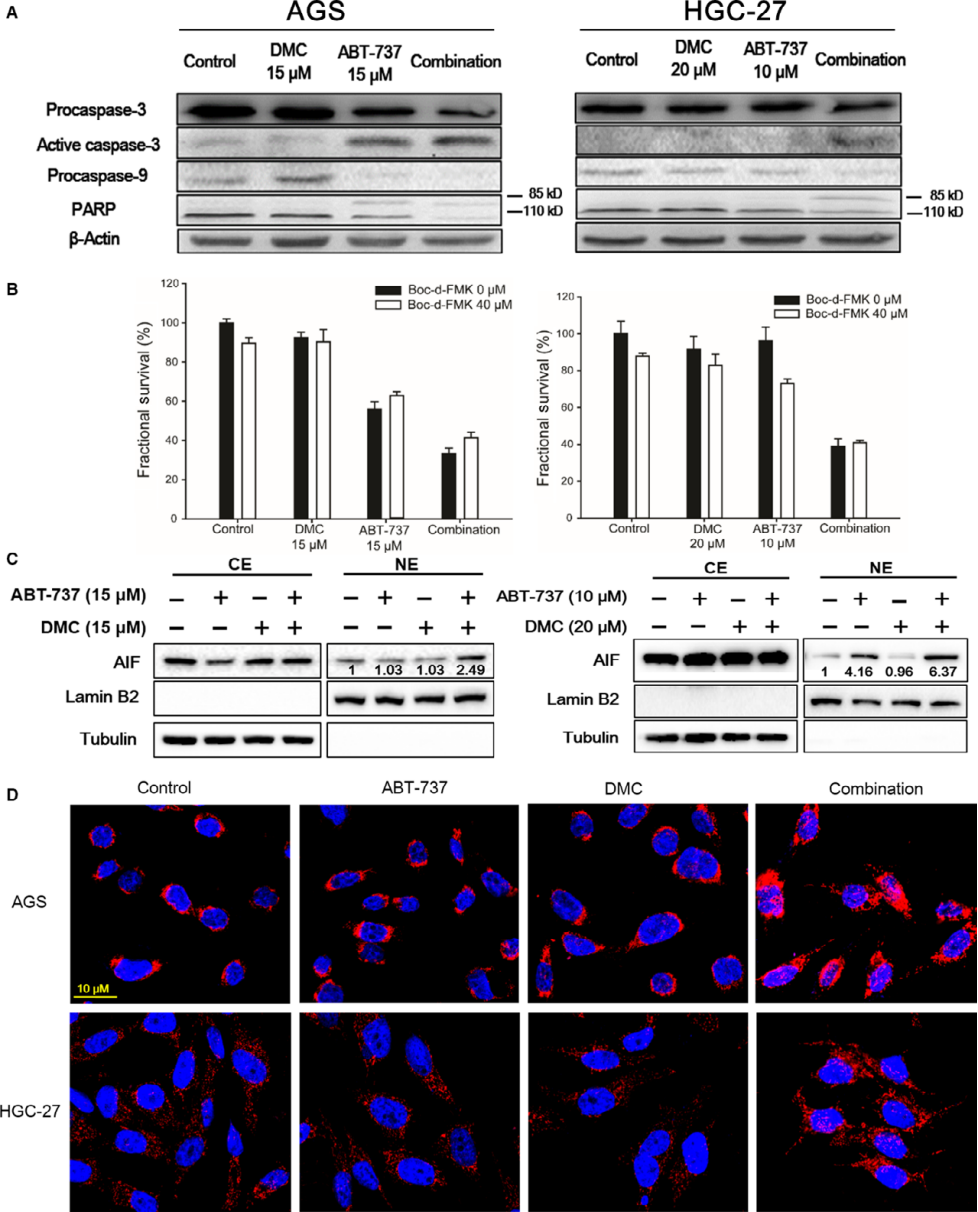
The combination of DMC and ABT‐737 triggered caspase‐independent apoptosis. (**A**) After treated with indicated compounds for 24 hrs, cells were lysed and proteins were analysed by Western blot. (**B**) Cells were pre‐cultured with Boc‐D‐FMK for 4 hrs before treatment. At the time of treatment, cells were co‐cultured with Boc‐D‐FMK and indicated compounds for another 8 hrs, followed by SRB cell viability assay. Each bar represented the mean ± S.D. (**C**) After treatment with DMC, ABT‐737 or the combination for 4 hrs, proteins from cytosolic and nuclear extracts were separated and analysed by Western blot assay. CE: cytosolic extracts; NE: nuclear extracts. (**D**) After treatment with DMC, ABT‐737 or the combination for 4 hrs, cells were fixed, incubated with primary‐ and secondary antibody, followed by confocal microscopy.

As shown in Figure 2, the combination treatment induced an obvious increased apoptosis rate in both AGS and HGC‐27 cells. We further explored the role of caspase pathway in this apoptosis. By examining the protein level of caspase‐3, caspase‐9 and PARP, it seemed that caspase pathway was moderately activated as a consequence of the combination treatment (Fig. 6A). However, the existence of pan‐caspase inhibitor Boc‐D‐FMK failed to overturn the cytotoxicity of either ABT‐737 or DMC mono‐treatment, or the combination (Fig. 6B). Using confocal microscopy to sub‐localize the intracellular position of AIF, we surprisingly found that the combination treatment dramatically promoted the translocation of AIF into cell nucleus, which was not observed with mono‐treatment under the same condition (Fig. 6D). In addition, we separated the proteins from cytosolic and nuclear extracts, and analysed the protein expression of AIF in both cytosolic and nuclear proteins (Fig. 6C). Lamin B2 and tubulin were used as internal protein loading controls for nuclear and cytosolic proteins respectively. After 4 hrs treatment, the combination treatment resulted in a 2.5‐ and 6.4‐fold increase in AGS and HGC‐27 cells respectively, demonstrating the accumulation of AIF in cell nuclear after combination treatment.

## Discussion

ABT‐737 is a typical small molecule inhibitor that has specific binding affinity to Bcl‐2, Bcl‐xL and Bcl‐w, but has low affinity to Mcl‐1. This characteristic of ABT‐737 has severely impeded its extensive applications as a single agent in further applications 22. An efficacious strategy to overcome the drawbacks or enhance the therapeutic effect of ABT‐737 is to combine anticancer compounds that obtained activity to abrogate Mcl‐1 protein level, and these combinations have been reported to achieve synergistic effect in various cancer cell lines 23, 24.

Previous studies have suggested that the occurrence of ER stress results in Mcl‐1 degradation *via* the up‐regulation of Noxa or Bak, both of which are BH3‐only proteins that have the E3 ligase affinity to Mcl‐1 25, 26. 2,5‐dimethyl‐celecoxib is reported to aggravate ER stress and up‐regulate the Noxa protein level 27. 2,5‐dimethyl‐celecoxib is an analogue of celecoxib and has no COX‐2 inhibitory effect 14, 28. In this study, DMC was found to have neglectable effect on intracellular PGE2 level comparing with celecoxib (Fig. 1D). Thus, the considerable cardiovascular side effects of celecoxib could be circumvented by using this celecoxib derivative 29. Notably, we found that DMC obtained stronger cytotoxicity than celecoxib when applied in combination with ABT‐737 in GC cells, which was consistent with previous published studies 17.

To explore the underlying mechanisms that DMC sensitize GC cells to ABT‐737, we first examined the effect of combination treatment on ER stress. Although the cytosolic calcium concentration increased to 6‐fold and 3‐fold in AGS and HGC‐27 cells respectively, ER stress‐mediated apoptosis marker CHOP was not found up‐regulated, indicating that the ER stress was to some extent enhanced, but not sufficient to induce apoptosis (Fig. 4) 30. We next measured the role of mitochondrial proteins in the combination treatment‐induced apoptosis. Mcl‐1 was the determinant of ABT‐737 drug sensitivity in a variety of cell lines 31. It was reported that Noxa determined the localization and stability of Mcl‐1 and consequently ABT‐737 sensitivity, and the expression level of Noxa was negatively correlated with Mcl‐1 in many cancer cell lines 25. In this study, the protein level of Noxa was unchanged after exposure to either ABT‐737 or DMC mono‐treatment or the combination. Moreover, other BH3‐only proteins such as Bim and Puma were not influenced by combination treatment.

Although mitochondrial membrane potential was depolarized and caspases were activated in the ABT‐737 and DMC combination‐induced apoptosis, it proved to be independent of the caspase cascade by using pan‐caspase inhibitor Boc‐D‐FMK. Instead, the existence of AIF and its translocation from mitochondria to the nucleus was indispensable to apoptosis, causing DNA fragmentation independently of caspase activity (Fig. 6C and D) 32, 33. Interestingly, single DMC or ABT‐737 treatment did not promote the translocation of AIF into cell nucleus in AGS cells, but only occurred while in combination treatment. In HGC‐27 cells, ABT‐737 treatment caused a 4‐fold increase in the nuclear accumulation of AIF, and combination treatment exaggerated the increase to 6‐fold (Fig. 6C). Establishing the connection between Mcl‐1 degradation and AIF translocation seemed necessary to fully understand the apoptosis regulation induced by Bcl‐2 inhibitors and also in combination, and would be done in the near future.

Taken together, this study found that combination treatment of ABT‐737 and DMC showed promising anti‐proliferative effect against GC cells. 2,5‐dimethyl‐celecoxib, as a non‐COX‐2 inhibitor, could obtain potential applications as an anti‐cancer compound, especially in combination therapy. More importantly, this study delineated that caspase‐independent apoptosis could be involved in Bcl‐2 inhibitors‐induced apoptosis, which could inspire other possible combination regimens to enhance the therapeutic effect of Bcl‐2 inhibitors.

## Conflict of interest

The authors declared no conflicts of interest.

## Supporting information


**Figure S1** The intensity of band Bax and Bcl‐2 in Figure 3C was quantitatively analysed, and the ratio of Bax/Bcl‐2 was graphed into bars. Each bar represented the mean ± S.D.
**Figure S2** The intensity of band Mcl‐1, Noxa and Puma in Figure 3C was quantitatively analysed, and the numbers were graphed into bars. Each bar represented the mean ± S.D.
**Figure S3** The intensity of band Bim and Bak in Figure 3C and ATF‐4 in Figure 4C was quantitatively analysed, and the numbers were graphed into bars. Each bar represented the mean ± S.D.
**Figure S4** The intensity of band Chop in Figure 4C and active caspase‐3, procaspase‐9 in Figure 6A was quantitatively analysed, and the numbers were graphed into bars. Each bar represented the mean ± S.D.
