# Supplementary material for: Dimethyl celecoxib sensitizes gastric cancer cells to ABT‐737 via AIF nuclear translocation
Source: J Cell Mol Med. 2016 Jul 4;20(11):2148–59. doi: 10.1111/jcmm.12913 (PMC5082400; doi:10.1111/jcmm.12913)

**Supporting Information**

**Dimethyl celecoxib sensitizes gastric cancer cells to ABT-737 via AIF nuclear translocation**

Bo Zhang^a,b,c^, Youyou Yan^a,b^, Yangling Li^a,b,c^, Dan Zhang^a,b,c^, Jianmei Zeng^d^, Linling Wang^d^, Mimi Wang^d^, Nengming Lin^a,b,c,d*^

*^a^*Laboratory of Clinical Pharmacology, Affiliated Hangzhou Hospital, Nanjing Medical University

*^b^*Laboratory of Clinical Pharmacology, Hangzhou Translational Medicine Research Center, Hangzhou First People's Hospital

*^c^*Department of Clinical Pharmacy, Hangzhou First People's Hospital

*^d^*Institute of Pharmacology, College of Pharmaceutical Sciences, Zhejiang Chinese Medical University

**Figure S1.** The intensity of band Bax and Bcl-2 in Figure 3C was quantatively analyzed, and the ratio of Bax/Bcl-2 was graphed into bars. Each bar represented the mean ± SD.


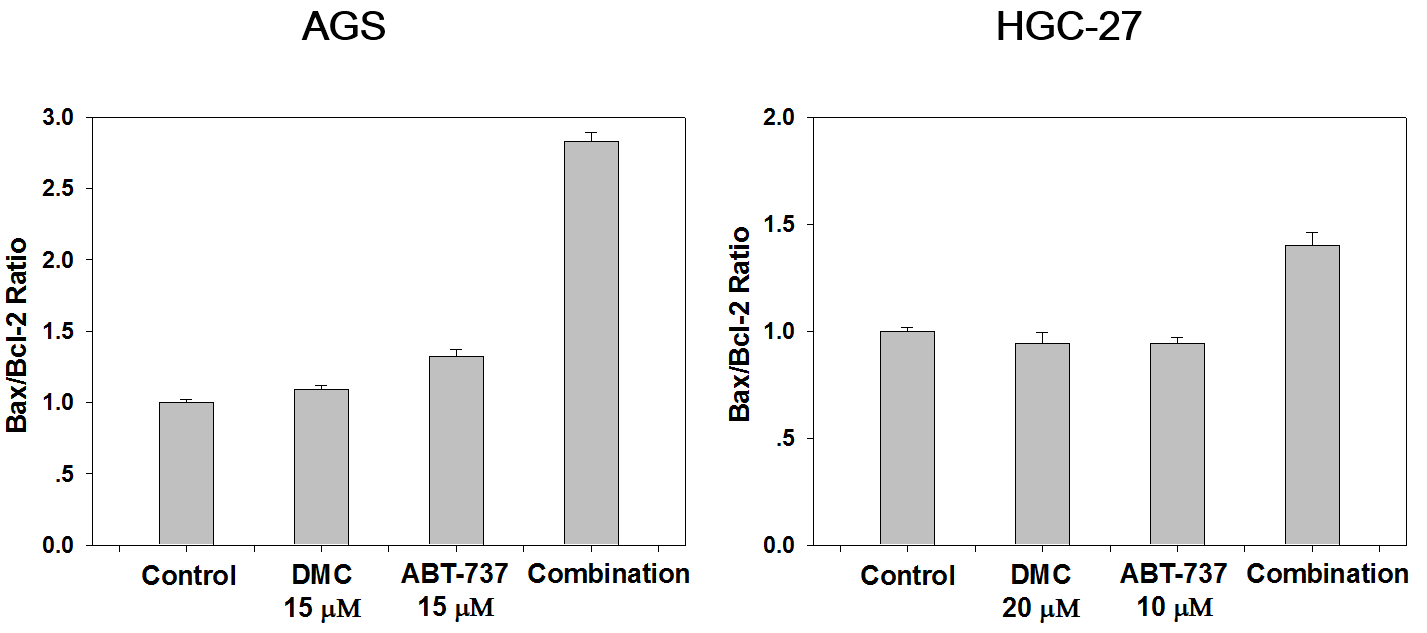


**Figure S2.** The intensity of band Mcl-1, Noxa and Puma in Figure 3C was quantatively analyzed, and the numbers were graphed into bars. Each bar represented the mean ± SD.


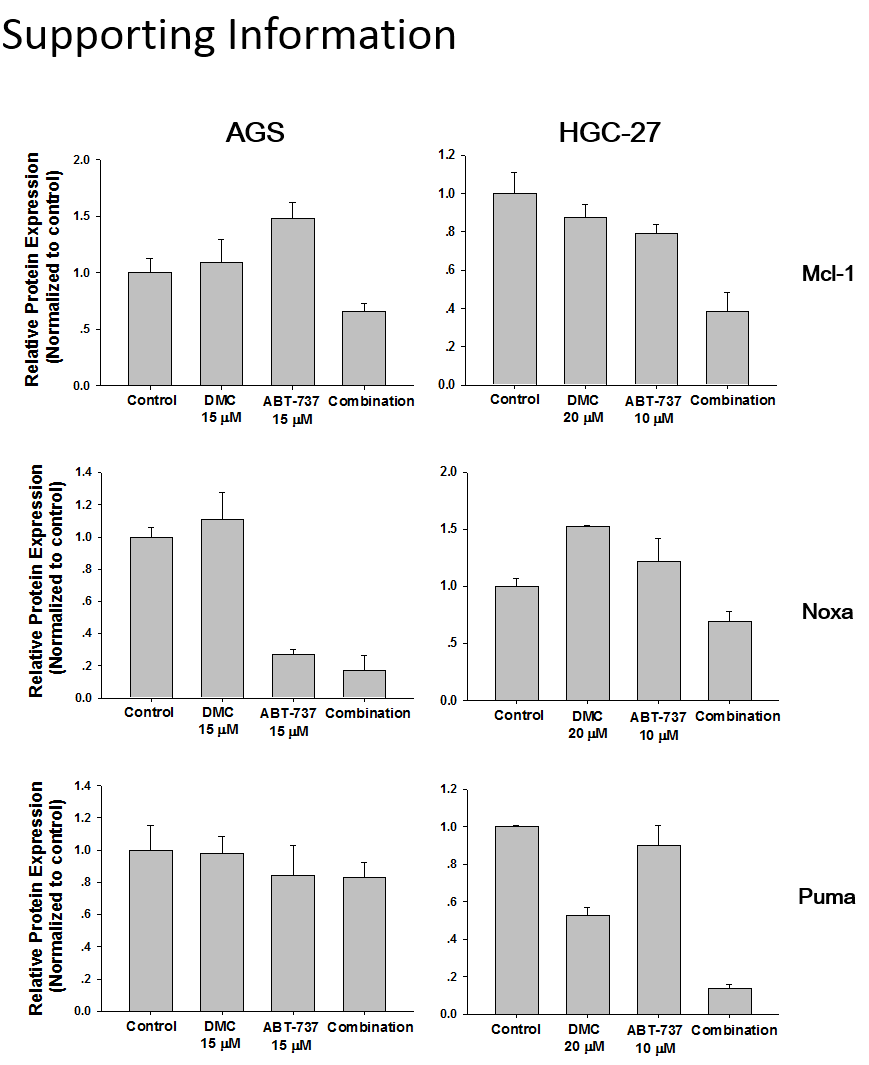


**Figure S3.** The intensity of band Bim and Bak in Figure 3C and ATF-4 in Figure 4C was quantatively analyzed, and the numbers were graphed into bars. Each bar represented the mean ± SD.


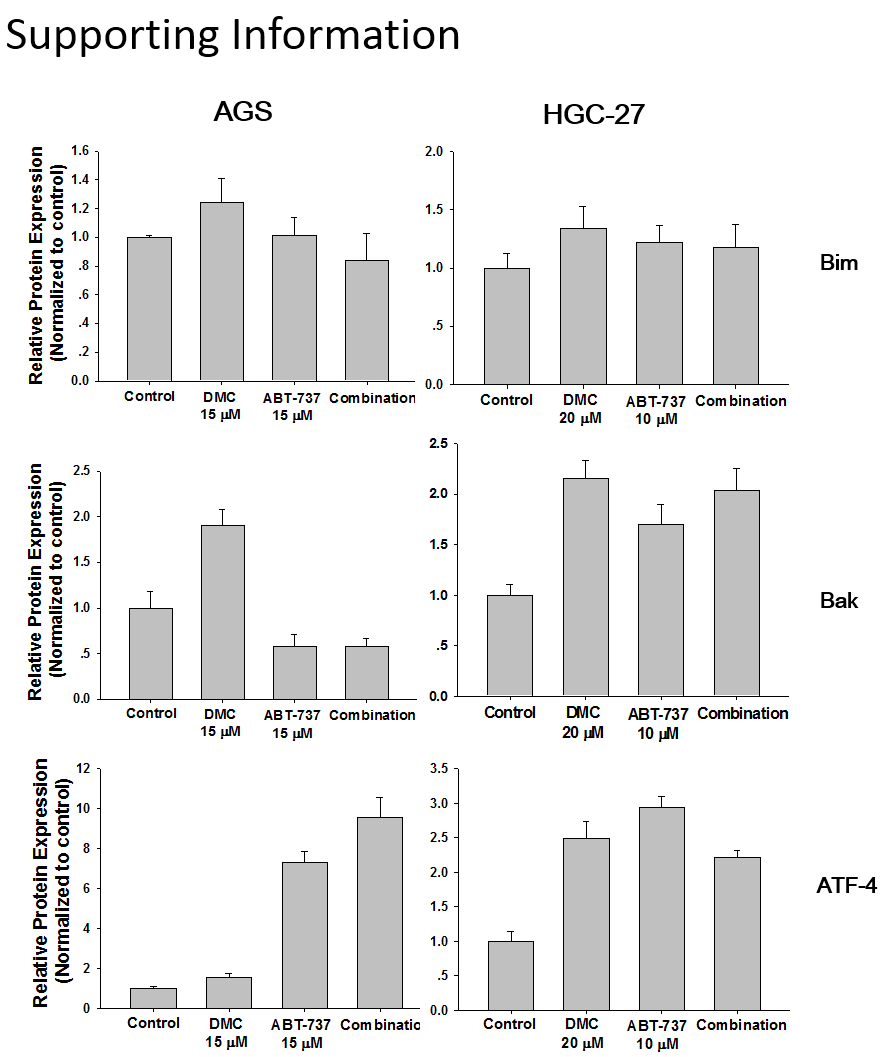


**Figure S4.** The intensity of band Chop in Figure 4C and active caspase-3, procaspase-9 in Figure 6A was quantatively analyzed, and the numbers were graphed into bars. Each bar represented the mean ± SD.


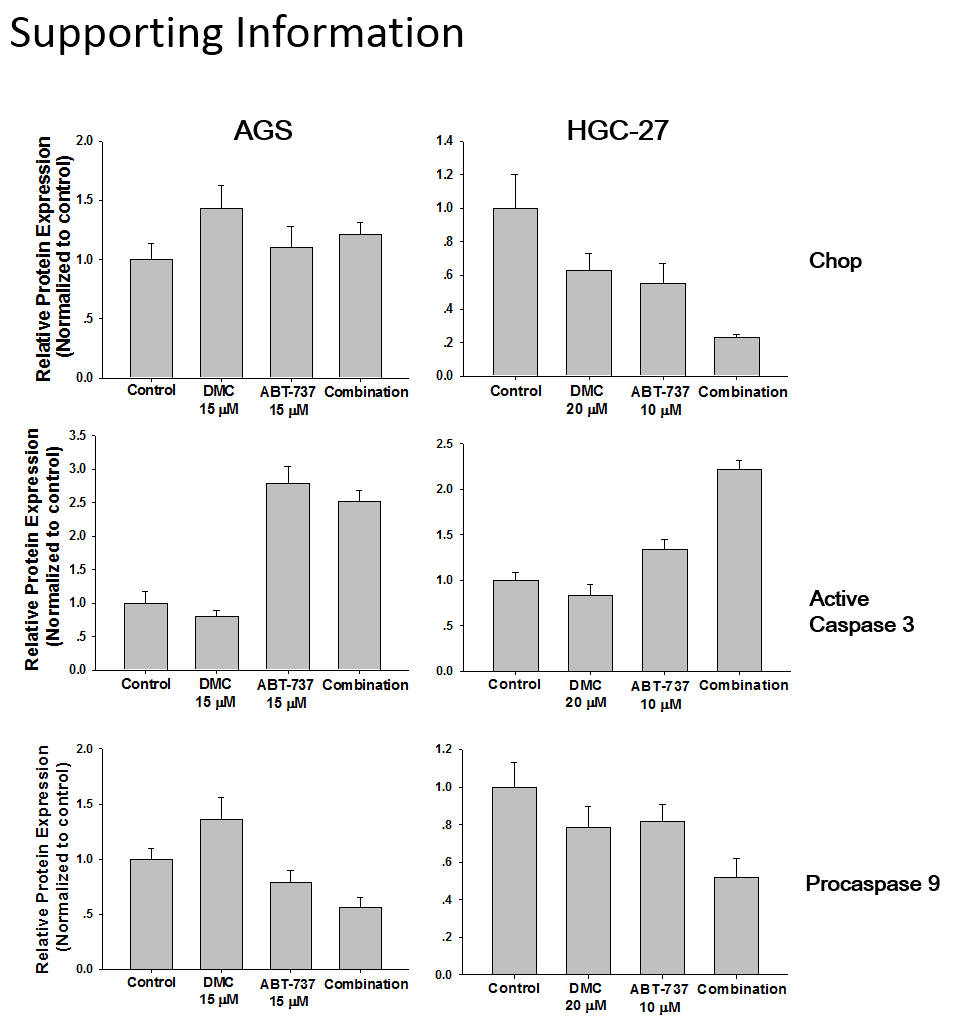

Supplement: Supplementary file 1 — Figure S1 The intensity of band Bax and Bcl‐2 in Figure 3C was quantitatively analysed, and the ratio of Bax/Bcl‐2 was graphed into bars. Each bar represented the mean ± S.D. Figure S2 The intensity of band Mcl‐1, Noxa and Puma in Figure 3C was quantitatively analysed, and the numbers were graphed into bars. Each bar represented the mean ± S.D. Figure S3 The intensity of band Bim and Bak in Figure 3C and ATF‐4 in Figure 4C was quantitatively analysed, and the numbers were graphed into bars. Each bar represented the mean ± S.D. Figure S4 The intensity of band Chop in Figure 4C and active caspase‐3, procaspase‐9 in Figure 6A was quantitatively analysed, and the numbers were graphed into bars. Each bar represented the mean ± S.D. [file JCMM-20-2148-s001.docx]
